# Aeromedical Transfer of Patients with Viral Hemorrhagic Fever

**DOI:** 10.3201/eid2501.180662

**Published:** 2019-01

**Authors:** Edward D. Nicol, Stephen Mepham, Jonathan Naylor, Ian Mollan, Matthew Adam, Joanna d’Arcy, Philip Gillen, Emma Vincent, Belinda Mollan, David Mulvaney, Andrew Green, Michael Jacobs

**Affiliations:** Royal Air Force Brize Norton, Oxfordshire, UK (E.D. Nicol, J. Naylor, I. Mollan, M. Adam, J. d’Arcy, P. Gillen, E. Vincent, B. Mollan, D. Mulvaney);; Royal Air Force Henlow, Bedfordshire, UK (E.D. Nicol, J. Naylor, I. Mollan, J. d’Arcy);; Royal Free London NHS Foundation Trust, London, UK (S. Mepham, M. Adam, M. Jacobs);; Level 2 Queen Elizabeth Hospital Birmingham, Birmingham, UK (A. Green)

**Keywords:** hemorrhagic fever, viruses, Ebola virus, infection control, patient transfer, patient isolation, aviation, aerospace medicine, United Kingdom

## Abstract

For >40 years, the British Royal Air Force has maintained an aeromedical evacuation facility, the Deployable Air Isolator Team (DAIT), to transport patients with possible or confirmed highly infectious diseases to the United Kingdom. Since 2012, the DAIT, a joint Department of Health and Ministry of Defence asset, has successfully transferred 1 case-patient with Crimean-Congo hemorrhagic fever, 5 case-patients with Ebola virus disease, and 5 case-patients with high-risk Ebola virus exposure. Currently, no UK-published guidelines exist on how to transfer such patients. Here we describe the DAIT procedures from collection at point of illness or exposure to delivery into a dedicated specialist center. We provide illustrations of the challenges faced and, where appropriate, the enhancements made to the process over time.

In the 1970s, the British Royal Air Force (RAF) was tasked with developing a portable isolation facility that could retrieve patients with infectious diseases. The original Vickers Isolator was manufactured at RAF Lyneham in 1982 and first used in 1985 (*1*). The Trexler Air Transport Isolator (T-ATI) system has since undergone modifications and improvements. Separate T-ATIs are owned by the Ministry of Defence (MOD) and the Department of Health, and both capabilities are operated by a dedicated MOD team that includes the medical operations directorate, the RAF Aeromedical Evacuation Coordination Cell (AECC), and the Deployable Air Isolator Team (DAIT). Civilian and military colleagues have trained and deployed together since 1996. Since 2012, this team has successfully transferred 1 patient with advanced Crimean-Congo hemorrhagic fever (*2*) and 5 patients with Ebola virus disease (EVD) (*3**–**5*), 2 with recrudescence or late neurologic complications (*6**,**7*). In addition, 5 patients with Ebola exposures, including 2 high-risk and 2 intermediate-risk exposures, have been transferred (*4**,**8**–**10*) (Table 1). 

**Table 1:** Aeromedical transfers of patients with confirmed and exposed viral hemorrhagic fever to the United Kingdom, 2012–2016*

Mission	Date	Infection	Origin	Patient signs/symptoms (stable/unstable)	Isolator type	Aircraft	Patient outcome
1	2012 Oct 4	CCHF	Afghanistan† (*1*)	Blood, diarrhea (unstable)	T-ATI	Lockheed C-130 Hercules	Died
2	2014 Aug 23	EVD	Sierra Leone (*4*)	None (stable)	T-ATI	Boeing C-17	Survived
3	2014 Dec 29	EVD	Sierra Leone† (*2*)	None (stable)	T-ATI	Lockheed C-130 Hercules	Survived
4	2015 Jan 29	Ebola exposure	Sierra Leone (*10*)	None	SI	Boeing C-17	Survived
5	2015 Jan 31	Ebola exposure	Sierra Leone (*10*)	None	SI	Boeing C-17	Survived
6	2015 Mar 13	EVD	Sierra Leone (*3*)	Diarrhea (stable)	T-ATI	Boeing C-17	Survived
		Ebola exposure	Sierra Leone (*10*)	None	SI		Survived
		Ebola exposure	Sierra Leone (*10*)	None	SI		Survived
7	2015 Mar 13	Ebola exposure	Sierra Leone (*10*)	None	SI	Boeing C-17	Survived
8	2015 Oct 9	EVD	United Kingdom (*5*)	Meningitis	T-ATI	Boeing C-17	Survived
9	2016 Mar 22	EVD	United Kingdom (*6*)	Late neurologic complications	T-ATI	Lockheed C-130 Hercules	Survived

*CCHF, Crimean-Congo hemorrhagic fever; EVD, Ebola virus disease; SI, stretcher isolator; T-ATI, Trexler Air Transport Isolator; UK, United Kingdom. †Aeromedical transfer originated within the United Kingdom.

In the United Kingdom, 2 high-level isolation units (HLIU) are primarily responsible for the care of patients with viral hemorrhagic fevers (VHFs): the Royal Free Hospital, London, and the Royal Victoria Infirmary, Newcastle. Both units use the Trexler patient isolator, in which patient care is provided within an isolation tent. End-to-end maximal patient containment from overseas to the receiving hospital and subsequent discharge is achieved through the T-ATI (Figure 1), which is designed to interface with the Trexler isolator. The T-ATI is transported on a suitable aircraft and from the airhead using a dedicated ambulance, the Jumbulance (Figure 2).

**Figure 1:**
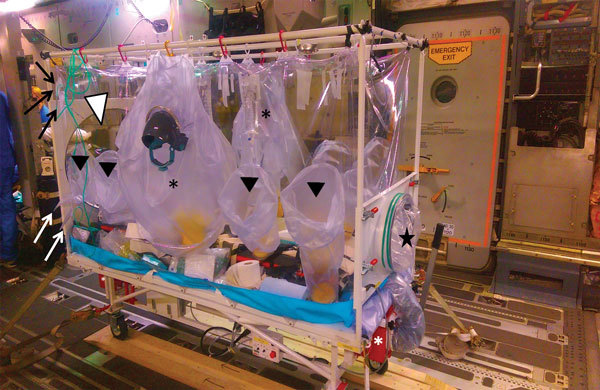
The Trexler Air Transport Isolator, a portable isolation facility used to transfer patients with serious infectious diseases. The sealed system is maintained under negative pressure by a HEPA-filtered ventilation system (red boxes, marked with white asterisk). Portable oxygen cylinders and tubing passed into the envelope through sealed delivery ports (black arrows) permit additional oxygenation of the patient. Additional ports allow cables for monitoring equipment and tubing for parenteral fluids or medication (white arrows). A half-suit on either side of the isolator (black asterisk) enables healthcare workers’ clinical access to the patient, and an additional half-suit can be fitted to the head of the patient for intubation (white arrowhead). Additional arm and glove ports along the side (black arrowheads) allow multiple workers to access the patient simultaneously. Two larger-bore disposable waste areas are available at the foot of the envelope (black star).

**Figure 2:**
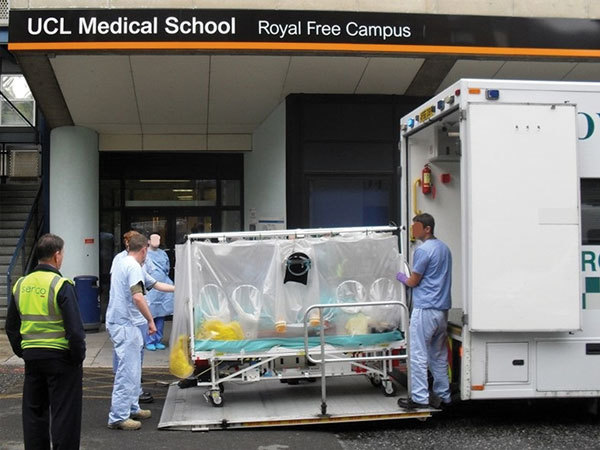
Dedicated road transport that can accommodate the Air Transport Isolator patient transport system, such as the Jumbulance shown, enables seamless end-to-end transfer from patient pickup to the destination facility.

## Aeromedical Evacuation of Patients with High-Consequence Infectious Disease

### The UK T-ATI

**Figure 3:**
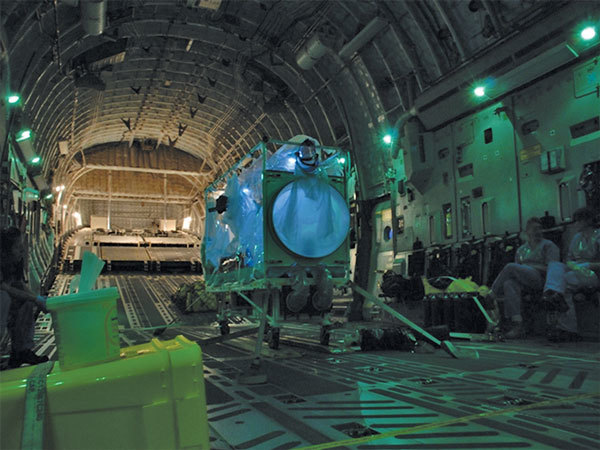
A single Trexler Air Transport Isolator patient transport system ready for use on a Boeing C-17 Globemaster transport aircraft.

**Figure 4:**
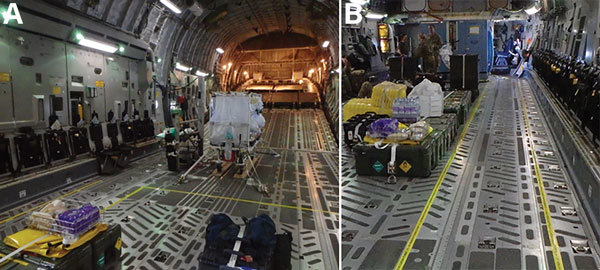
Demarcation of clean and dirty zones during use of the Trexler Air Transportable Isolator patient transport system on a Boeing C-17 Globemaster transport aircraft. A) Yellow lines clearly demarcate clean and dirty zones as required for transporting both confirmed and exposed viral hemorrhagic fever case-patients. B) For exposed patients, the demarcation zone should extend to a corridor leading to isolated toileting and comfort facilities.

There are 2 basic designs of Air Transport Isolator systems, closed and open, both of which offer maximum containment of highly infectious biologic agents. Open systems provide a portable isolation facility large enough for both the patient and attending medical staff wearing personal protective equipment (PPE), whereas closed systems separate the patient from attending physicians (*11*). Several commercially available closed isolation systems have been developed from chemical, biologic, radiologic, and nuclear biodefense funding streams. These are stretcher isolators (S-ATI) designed to manage patients exposed to infectious agents, as opposed to patients with symptoms of infectious diseases. Because S-ATI are small, their capability to support the transfer of sick patients is limited. The United Kingdom uses a larger closed T-ATI system for the transfer of infected patients (Figure 1), a design that provides patient comfort and medical care while maintaining containment for the duration of the transfer mission. These systems have been used on Lockheed Martin C-130 and Boeing C-17 Globemaster (Figures 3,4) aircraft, enabling rapid delivery of the T-ATI and clinical team to both standard and austere landing strips at great distance from the United Kingdom.

The sealed T-ATI system includes an ATI frame and disposable envelope. It is maintained under negative pressure by a HEPA-filtered ventilation system that uses aircraft power when emplaned and battery power when outside the aircraft, while the cabin pressure is maintained at a standard cabin altitude of 8,000 feet. Clinically, this system maintains the arterial oxygen hemoglobin saturation at ≈90% in healthy patients, even inside the negative-pressure envelope. Additional oxygenation is possible through the use of portable oxygen cylinders and tubing passed into the envelope through sealed delivery ports. Cables for monitoring equipment, such as electrocardiogram electrodes, pulse oximeters, and blood pressure sensors, also pass through the ports, as does tubing for parenteral fluids or medication.

**Figure 5:**
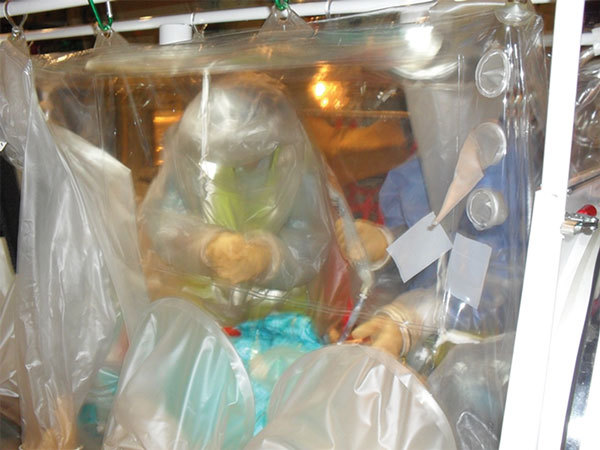
Larger Trexler Air Transport Isolator patient transport systems enable care providers to access a patient via half-suits along the side of the patient; however, manual dexterity is severely impaired

Clinical access consists of a half-suit on either side of the T-ATI; an additional half-suit can be fitted to permit access to the head and neck of the patient for intubation, if required (Figure 5). Arm and glove ports along the side of isolator enable multiple staff to access the patient simultaneously (Figure 1). At the foot of the envelope are 2 clinical waste disposal areas. Medical equipment likely to be needed for the management of the patient during the flight, such as intubation equipment, bag-valve mask, suction units, and intravenous access equipment, must be placed within the envelope before sealing the unit.

Logistical challenges overseas make moving the ATI away from the aircraft impractical, so the patient is usually moved to the airhead using appropriate and proportional infection prevention control (IPC) measures tailored to the location (*12*). Aeromedical personnel, who are fully trained in the use of PPE and decontamination, assist the patient into the T-ATI before boarding. Once the T-ATI is sealed, the patient’s care does not require staff PPE. Upon the aircraft’s return to the United Kingdom, a dedicated T-ATI Jumbulance is positioned at the receiving airbase to transport the T-ATI and to minimize the risk for external contamination.

### Guidance on the Air Transfer of VHF

Before 2014, UK guidance focused on the public health response to inadvertent importation of cases of VHF on domestic aircraft (*13*); some US guidance existed on the use of closed isolator procedures (*14*). In response to the 2014–2016 Ebola crisis in West Africa, the US Centers for Disease Control and Prevention (*15*), Canadian Critical Care Society (CCCS) (*16*), and European Centre for Disease Prevention and Control (*11*) have published patient transport guidelines. As the sole UK provider of air transfer of highly infectious patients, the MOD has extensive standard operating procedures for the retrieval of both civilian and military patients with EVD. In 2014, the United States, through license with Phoenix Air (*17*), developed an open system with VHF capability and now has 2 options for transporting highly contagious patients: in single-patient isolator units that are similar to the T-ATI (the Phoenix Air Airborne Biologic Containment System [*18*]) or in fully contained medical transport units (developed by MRI Global, with support from the US Department of State and the Paul G. Allen Ebola Program [*19*]).

### Case Referral

The decision to evacuate patients with highly infectious diseases from their point of infection or diagnosis to the United Kingdom is a complex process that considers the clinical, public health, and political contexts. The clinical decision is made between the clinical lead in-country, the on-call HLIU physician at the Royal Free Hospital, and the on-call RAF medical consultant, with additional advice from the RAF public health lead as required. The flight details are planned in conjunction with AECC.

The initial call to the United Kingdom referring confirmed or suspected VHF cases or a high-risk exposure is taken by either the National Health Service England Emergency Preparedness Resilience and Response Duty Officer, the RAF, or the HLIU, Royal Free Hospital (London, UK). The transfer is coordinated by National Health Service England Emergency Preparedness Resilience and Response and approved and enabled through Cabinet Office Briefing Room meetings and Ministers of State. Once approved there is a cross-governmental process to coordinate local and national UK government, the receiving HLIU, MOD Med Ops, and RAF AECC staff.

### DAIT Activation

Ultimately, the transfer of a patient is authorized only if it is deemed in the best interest of the patient, after which AECC activates the DAIT. After receiving the order to deploy, the DAIT personnel convene at RAF Brize Norton (Oxfordshire, UK) to plan and execute the mission, and communicate with receiving hospitals, specialist ambulance services, police and security services, government agencies, ministerial offices, and multiple agencies involved in flight planning and diplomatic clearances (*20*). For T-ATI transfers, the Jumbulance is prepositioned to the receiving airfield to enable a seamless, contained transfer of the patient within the T-ATI by road from the aircraft to the receiving hospital. The length of the mission, from start to the return of the aircraft, is often >24 hours.

For asymptomatic exposure cases in which isolation of the source patient is not deemed necessary at the outset, a T-ATI is brought aboard to ensure immediate appropriate isolation if symptoms develop in flight. The DAIT is responsible for the planning, preparation, and conduct of the clinical aeromedical recovery, from the point of origin to the destination hospital, in a manner that protects the aircrew, aeromedical staff, and local populations while providing high-quality clinical care to the patient. For flights that require overflying other nations, the DAIT routinely seeks diplomatic clearance. In the cases we described, either there was no requirement for clearance (over-sea flight route to Sierra Leone) or the flight was within UK airspace.

The DAIT is continuously available, held at a high level of readiness, with DAIT staff on call for a month at a time on average every 3 months. At times of high operational demand, the call period may be longer or the frequency of service higher. When on call, DAIT staff convene for monthly PPE/T-ATI training and refresher training, reinforcing the team ethos required for these arduous and emotive missions and ensures skill retention.

### UK DAIT Team Composition

The team composition is tailored to the requirements of the specific mission. To move a single highly contagious patient requires 12 personnel, including IPC nurses, an RAF consultant physician and anesthetist, a civilian infectious diseases consultant from the Royal Free HLIU team, medical assistants, a medical and dental support system technician to assess and monitor T-ATI integrity, and a medical support officer, who acts as a liaison with the host nation nonclinical staff, ensures involvement of legitimate personnel only, and minimizes press access. Oversight of the whole mission is provided by a team leader/flight director who remains removed from direct clinical care and allows the clinical team to focus solely on the patient’s needs without distraction. Additional staff are considered for flights involving >2 patients, depending on the clinical scenario, and the United Kingdom maintains the capability to activate 2 teams concurrently if required.

### Deployment of the DAIT

Before the DAIT departs the United Kingdom, and before equipment is loaded onto the airplane, final preparations ensure that all potential in-flight needs have been addressed, and that all personnel have appropriate travel and health documentation (visas, antimalarial medication, and immunization evidence). As part of the risk assessment conducted in the United Kingdom, an advance military reconnaissance team may conduct an initial assessment with the local treatment facility staff in the country of patient origin, with respect to the patient and the clinical context. If in-country retrieval by the DAIT can be avoided, the benefits for both the DAIT and patient include minimized safety and security risks in volatile regions, time spent in PPE, and fatigue on the return flight. Alternatively, the DAIT can split into 2 parts on arrival: a reconnaissance team to assess the patient, and the main party to prepare the T-ATI, decontamination area, and aircraft.

The reconnaissance team, usually consisting of the clinical lead, civilian ID consultant, an infection control nurse, and a medic, performs the initial assessment of the patient, either at the host hospital or preferably at the airhead. Ideally, the team conducts the assessment wearing full PPE consistent with UK national guidance (*18*) for confirmed cases. However, local sensibilities need to be considered and may dictate that a more practical compromise is required while minimizing risk to medical attendants and maximizing patient safety; thus, the DAIT reconnaissance team may alter PPE only if appropriate. An example is evacuating a foreign aid worker from a facility that is the only option for local patients. The team must quickly and efficiently assess the patient’s clinical and mental status for entering a T-ATI, as well as assess the potential effects of the hypobaric, hypoxic aeromedical environment, the overall safety of the transfer, and the underlying conditions on the ground. Once at the airhead, the team usually conducts a final assessment immediately before transferring the patient to the ATI.

As with any aeromedical evacuation, a full risk assessment that considers the safety of the patient, team, aircrew, and aircraft must be performed before embarkation. Whereas it might appear to be futile to cancel a mission at this stage, if the risk to transfer is deemed too high (such as in advanced disease), the flight environment deemed unwise (high risk for hypoxia or long transfer with limited available intervention), or the risk for deterioration too great, the reconnaissance team and team leader must make this judgment call. Alternatively, the team may deem a case less infectious, which requires a lower level of isolation (for example, management of exposure to contacts). The team must also consider interventions, such as cannulation or catheterization, that may be easier to perform while in PPE on the ground before boarding the plane.

Meanwhile, the main team positions the T-ATI and sets up the decontamination area for the reconnaissance team. The aircraft is set up with demarcated clean and dirty zones and, for VHF exposure cases in which the T-ATI is not deemed necessary at the outset of the flight, an identified corridor (Figure 4, panels A, B) for the patient to access a dedicated area for eating and toileting. These measures separate the patient from all but immediate clinical staff and ensure safe IPC measures in the event of clinical deterioration.

**Figure 6:**
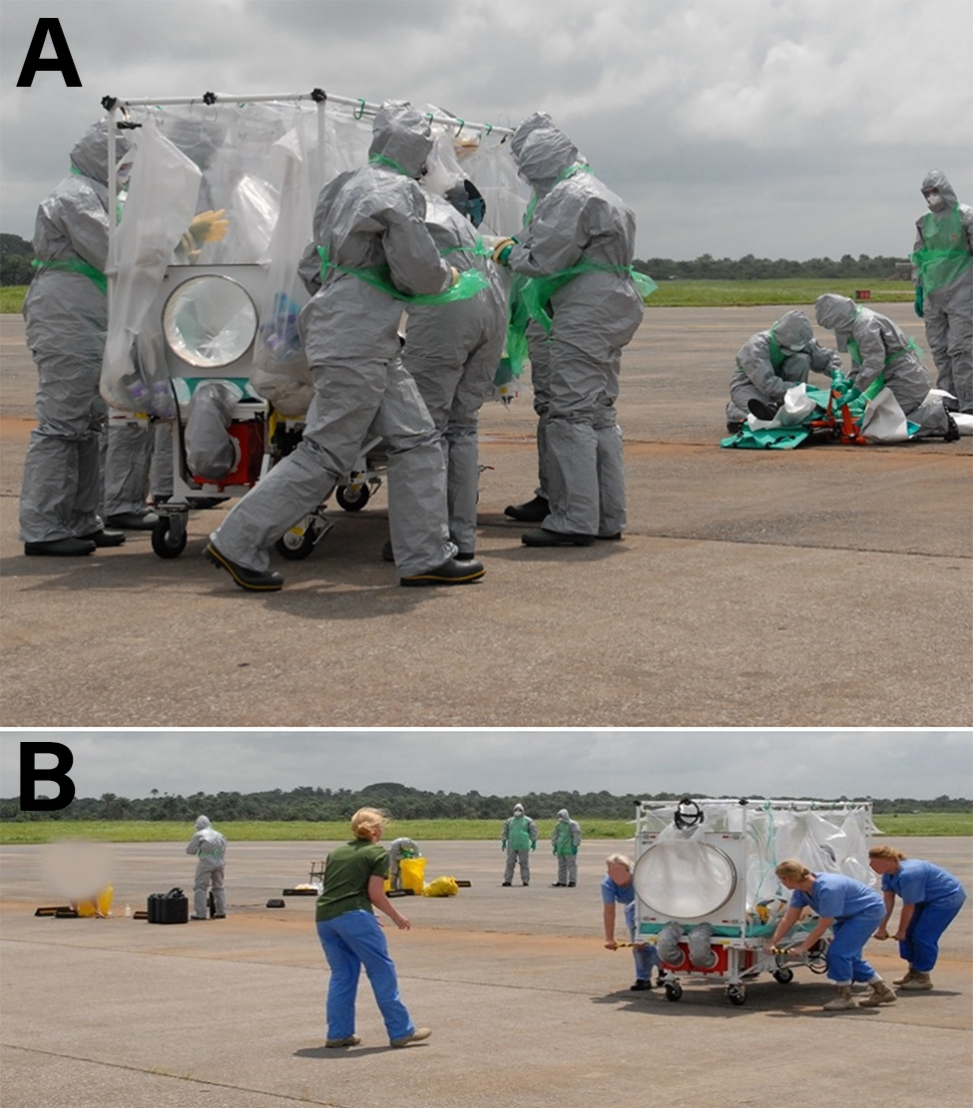
The Deployable Air Isolator Team lead, a senior infection and prevention control nurse, is responsible for overseeing the preparation of the Air Transport Isolator patient transport system on the ground (A), the transfer of the patient into the isolator, and the safe transfer of the patient onto the aircraft by the main team while the reconnaissance team performs their decontamination drills (B).

For confirmed cases, the DAIT leader coordinates the safe loading of the patient into the T-ATI and its subsequent loading onto the aircraft. This process is followed by the reconnaissance team decontamination, with assistance from trained RAF regiment staff for PPE doffing (Figure 6) and clinical waste management.

### Delivery of In-Flight Care

The challenges of delivering in-flight clinical care to a patient within the T-ATI are substantial; time needed to enter the half-suits, limited visibility, reduced manual dexterity, and aircraft noise and vibration make any action beyond observations and simple intervention difficult. Drugs and parenteral fluids for hydration or to replace electrolyte abnormalities are administered through closed circuits and longer lines with larger volume flushes to minimize exposure risk and permit rapid administration from outside the isolator. In extreme cases, if a patient deteriorates, the team could consider administering drugs such as vasopressors but would need to deliver them through existing venous access (either peripheral or central). If the patient’s condition includes agitation, anxiety, or confusion, the team can offer anxiolytic or sedative therapy and should be prepared to manage a potentially compromised airway. To date, most patients transferred have not been critically ill, with the exception of the Crimean-Congo hemorrhagic fever transfer from Glasgow to London in October 2012. Unless it is critical to move an unstable patient, or one who is expected to significantly deteriorate, the patient may be best served with local medical care. Nonmedical requirements, such as hydration and food, must be considered before patient isolation, and the risk from human waste can be minimized by using containers with absorbent powders or gels that solidify fluids. 

The DAIT team members do not need to remain in full PPE once the patient is inside the isolator. Rotating the nursing team every hour on the inbound flight minimizes fatigue and ensures delivery of quality care. A death in flight is managed with standard procedures, which vary depending on the jurisdiction of the flight.

### Patient Delivery in the United Kingdom

**Figure 7:**
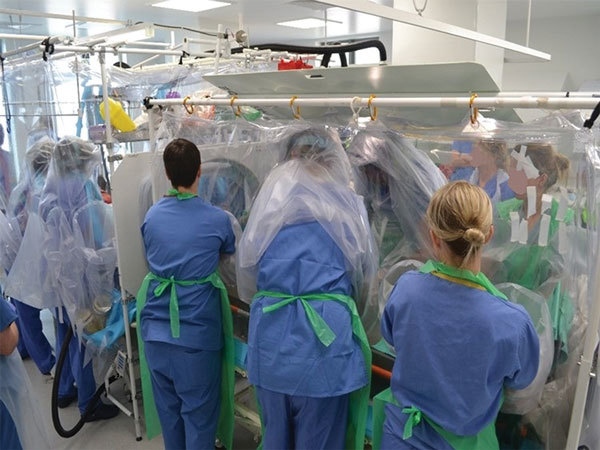
Isolator–isolator transfer is the safest means of transfer for patients with serious infectious diseases and requires practice in dedicated training exercises, as shown.

The aircraft always lands as close to the receiving hospital as possible to reduce the transfer time; close coordination between the airport staff, police, and ambulance services ensures a secure arrival process. For cases of exposure only, transfer in a standard ambulance is often appropriate without the need for PPE; for confirmed VHF cases in the T-ATI, Jumbulance transfer with a police escort minimizes delay and ensures adequate security. Coordinating arrival at the specialist center (Figure 2) permits securing and clearing the transfer route to allow smooth passage to either the patient isolator (Figure 7) in the HLIU or a negative-pressure room.

After delivery of the patient to the receiving unit, the envelope, T-ATI frame, and Jumbulance require decontamination. After the HLIU staff’s thorough assessment of the envelope to determine if any breaches have occurred, the T-ATI envelope and frame are fumigated with vaporized hydrogen peroxide (*21*). The envelope is then collapsed, autoclaved, and disposed as conventional clinical waste onsite. After the removal of patient’s clinical waste, DAIT and HLIU personnel decontaminate the Jumbulance. Staff then shower and don fresh scrubs. The decontamination of the Jumbulance takes a few hours, and the T-ATI frame is returned within 7 days for reuse. Twice-yearly training between the RAF and HLIU includes the review of protocols and equipment.

Aeromedical evacuations are often long, with DAIT staff working for 24–36 hours. Aircrew manage aeromedical evacuations, including those using the T-ATI, as for any other flight operation, with consideration of crew hours in accordance with standard procedure. During the 2014–2016 Ebola outbreak, a typical move to Sierra Leone would involve activating the team with a “6-hour wheels up” policy from point of activation. Within these 6 hours, the DAIT would travel to RAF Brize Norton and undertake equipment checks, aircrew would conduct flight preparation, and AECC staff would undertake ongoing communications and diplomatic clearances. The flight time to Sierra Leone was 8–10 hours each way, and crews spent up to 3 hours on the ground. After the return flight, the road transfer and handover to the receiving unit typically took 4 hours, including recovery of the DAIT to RAF Brize Norton.

## Discussion

**Figure 8:**
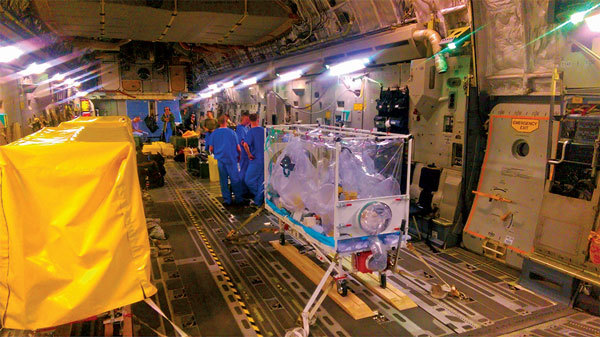
Multiple Air Transportable Isolator patient transport systems on a single aircraft (Boeing C-17 Globemaster). A single isolator is set up for the confirmed viral hemorrhagic fever case-patient; 2 additional isolators (left, covered) are available for the 2 exposed patients should they deteriorate or become symptomatic in flight.

The 2014–2016 Ebola outbreak in West Africa reaffirmed the requirement for aeromedical evacuation capability (a combination of both the appropriate equipment and suitable expert personnel trained in both the management of VHF and AE) for highly infectious diseases. During 2014 and 2015, the DAIT successfully transferred 5 patients with confirmed VHF and 5 with Ebola virus exposure. Each mission provided an opportunity to refine policy and procedure (Table 2), with resulting improvements to policy, training, and equipment. A larger number of T-ATIs and more efficient VHF decontamination have shortened turnaround time between transports and enabled versatility through accommodation of multiple T-ATIs and exposure cases (Figure 8). Information transfer is more robust between clinical staff at all levels and among the aircrew, logistic support teams, and senior military, civil, and political stakeholders. A clear organizational structure supports rapid decision making from the highest political level down to service delivery.

**Table 2:** Limitations and challenges in Deployable Air Isolator Team missions and subsequent enhancements, United Kingdom*

Limitations and challenges	Enhancements
Mission 1: Advanced CCHF in Glasgow—400-mile transfer to HLIU London (*2*)	
UK cross-governmental communication and media interest: Identifying the correct persons within the relevant UK and Scottish government departments to authorize the substantial costs involved was challenging because the Department of Health had restructured and NHS England formed with a loss of critical contact details. The coordination of the clinical transfer, with limited clinical experience of VHF and lack of standard operating procedures, and concurrent management of the extensive media interest, was time consuming and, at times, risked distraction from patient care, particularly for the lead clinician.	• Allocation of roles out with the front-line team for liaison with and arranging authorization by governmental departments. • Addition of Liaison Officer to manage extensive media interest (*3**–**5**,**8*) and minimize intrusion on patient dignity. • RAF anesthetic consultant for support of assessment, transfer and airway management such as in the event of neurologic compromise (*2**,**6*). • Civilian infectious diseases expert to allow an independent critical eye to assess and modify DAIT procedures and equipment. • Review of service level agreement between Department of Health and MOD for national air transfer (only international prior provision existed). • Recognition that road transfer in standard VHF PPE (*20*) posed increased risk.
Mission 2: Decontamination	
Before 2014, the T-ATI was decontaminated using formaldehyde before it was incinerated. This relatively slow and intensive process was potentially limited by lack of access to the whole T-ATI frame and by requiring physical cleaning by humans, increasing risk to staff.	• A new vaporized hydrogen peroxide protocol has enabled much faster turnaround time and safer T-ATI decontamination (*21*).
Missions 2 and 3: Environmental effects on working in PPE	
Heat and humidity while wearing chemical-resistant Tychem F PPE suits (Figure 6) posed challenges in Sierra Leone, while steamed-up goggles and sweat-filled gloves resulted in the loss of vision and dexterity. Gusting wind made decontamination and equipment containment difficult, compounding communication difficulties due to PPE and aircraft noise. Conversely, at Glasgow International Airport, Glasgow, Scotland, UK, near-freezing temperatures were experienced during the T-ATI transfer and decontamination procedures, and the hours of darkness presented visibility problems when working in PPE.	• Subsequent mission staff numbers, previously kept low to minimize VHF exposure, were revised upward for confirmed cases, and the use of lighter Tychem B/C suits offered the same protection.
Missions 4 and 5: Needle-stick exposure	
The DAIT were deployed to Sierra Leone to assess and transport HCWs who sustained a needle-stick injury while working in an Ebola treatment center (*4**,**8**,**9*). An in-country risk assessment permitted HCWs to return to the United Kingdom as standard aeromedical evacuations with DAIT as escorts, after initially being deemed too high risk to travel on a commercial airline. A T-ATI was kept on standby in case of clinical deterioration.	• In-country risk assessment modified the role of the DAIT to provide standard aeromedical evacuation with T-ATI on stand-by for those with high-risk exposure rather than confirmed EVD. • Civilian infectious diseases consultant enabled more rapid access to advanced EVD treatments for the injured HCWs.
Mission 6: Multiple patients on one platform, one confirmed in T-ATI and 2 exposed contacts with T-ATI on standby (Figure 5)	
Three military HCW exposed to Ebola were returned from the Ebola Treatment Centre, Kerrytown, Sierra Leone, alongside a confirmed Ebola case-patient. After an in-country risk assessment, 3 T-ATIs were flown on a single C-17 airframe (Figure 5) to Sierra Leone (*4*). Two exposed HCWs joined the RAF flight to the HLIU, Royal Free Hospital; 2 were flown back 48 h later by commercial flight to the HLIU, Royal Victoria Hospital in Newcastle.	• Team augmented to 22 personnel for 3 T-ATI. Marked out floating clean/dirty line through aircraft should all 3 T-ATI be used.

*CCHF, Crimean-Congo hemorrhagic fever; DAIT, Deployable Air Isolation Team; EVD, Ebola virus disease; HCW, healthcare worker; HLIU, high-level isolation units: MOD, Ministry of Defence; NHS, National Health Service; PPE, personal protective equipment; RAF, Royal Air Force; T-ATI, Trexler Air Transport Isolator; VHF, viral hemorrhagic fever.

We expect this resource to continue to evolve, shaped by future collaborations. The greatest challenge that emerged after the 2014–2016 Ebola epidemic is maintaining corporate knowledge and expertise; to ensure competence and confidence managing VHF requires continuous training. Regular exercises that test not only individual staff but the whole system, with particular focus on interagency collaboration, are required. Most patients transferred during the Ebola outbreak had been asymptomatic or were in the early stages of disease; innovation in closed T-ATI development is required to optimize the care provided to symptomatic patients, such as improved ergonomic design to make clinical intervention as simple as possible and PPE development to facilitate easier patient management. There is an ongoing role for single-person isolation units and portable biocontainment units that can provide for multiple patients and providers in a more functional medical unit. The clinical disadvantages of the current T-ATI include its size (it is not transportable on helicopters, or without a dedicated Jumbulance) and reduced dexterity that challenges staff in delivering optimal patient care.

For the past 40 years, the UK T-ATI system has provided end-to-end biologic containment from overseas site to specialist unit. Since 2012, a total of 8 successful missions have provided learning opportunities that have greatly improved the service. The system allows for a highly specialized capability, providing both moral support to those combating the disease overseas and safe transfer of potentially isolated cases to or within the United Kingdom (*22*).

Although the United Kingdom expects to continue to use the T-ATI, more advanced systems have been developed that are smaller and more versatile and may also provide easier clinical access to the patient. The United Kingdom is not currently looking to use larger transportable medical units. If the mission changes, the T-ATI, developed in the United States, remains an alternative solution if the numbers of patients requiring transfer increase or there is a demand to provide a higher level of care to VHF patients. It is possible that a greater future threat comes from airborne infectious disease; the flexible, adaptable, responsive, and safe capability of T-ATI and DAIT allows the transport of patients with highly transmissible airborne diseases back to the United Kingdom if required.

Because many nations do not have an aeromedical evacuation facility, it is unclear whether the RAF should repatriate patients to non–United Kingdom destinations. If it should, considerations include logistics of patient delivery from airstrip to the receiving unit (either in the T-ATI or in PPE); appropriate in-hospital transfer; and safe clinical waste and T-ATI envelope disposal and decontamination. 
